# Neuroprotective effect of L-DOPA-induced interleukin-13 on striatonigral degeneration in cerebral ischemia

**DOI:** 10.1038/s41419-024-07252-x

**Published:** 2024-11-22

**Authors:** Eunhae Jeon, Myeong-Seong Seo, Enkhmaa Lkhagva-Yondon, Yu-Ree Lim, Seung-Woo Kim, Yu Jeong Kang, Jun Seok Lee, Byoung Dae Lee, Rayul Wi, So-Yoon Won, Young Cheul Chung, Eun S. Park, Eunhee Kim, Byung Kwan Jin, Myung-Shin Jeon

**Affiliations:** 1 Translational Research Center, Inha University Hospital, Incheon, Republic of Korea; 2 Program in Biomedical Science & Engineering, Inha University, Incheon, Republic of Korea; 3 Department of Biomedical Sciences, College of Medicine, Inha University, Incheon, Republic of Korea; 4 Department of Physiology, School of Medicine, Kyung Hee University, Seoul, Republic of Korea; 5 Department of Biochemistry & Molecular Biology, School of Medicine, Kyung Hee University, Seoul, Republic of Korea; 6 Department of Predictive Toxicology, Korea Institute of Toxicology 1, Human and Environmental Toxicology, University of Science and Technology, Daejeon, Republic of Korea; 7 Vivian L. Smith Department of Neurosurgery, McGovern Medical School, The University of Texas Health Science Center at Houston, Houston, TX USA

**Keywords:** Neurological disorders, Cell death in the nervous system

## Abstract

Levodopa (L-DOPA) treatment is a clinically effective strategy for improving motor function in patients with ischemic stroke. However, the mechanisms by which modulating the dopamine system relieves the pathology of the ischemic brain remain unclear. Emerging evidence from an experimental mouse model of ischemic stroke, established by middle cerebral artery occlusion (MCAO), suggested that L-DOPA has the potential to modulate the inflammatory and immune response that occurs during a stroke. Here, we aimed to demonstrate the therapeutic effect of L-DOPA in regulating the systemic immune response and improving functional deficits in mice with ischemia. Transient MCAO led to progressive degeneration of nigrostriatal dopamine neurons and significant rotational behavior in mice. Exogenous L-DOPA treatment attenuated the striatonigral degeneration and reversed motor behavioral impairment. Notably, treatment with L-DOPA significantly increased IL-13 but reduced IFN-γ in infarct lesions. To investigate the role of IL-13 in motor behavior, we stereotaxically injected anti-IL-13 antibodies into the infarct area of the mouse brain one week after MCAO, followed by L-DOPA treatment. The intervention reduced dopamine, IL-13, and IL-10 levels and exacerbated motor function. IL-13 is potentially expressed on CD4 T cells, while IL-10 is mainly expressed on microglia rather than astrocytes. Finally, IL-13 activates the phagocytosis of microglia, which may contribute to neuroprotection by eliminating degenerating neurons. Our study provides evidence that the L-DOPA-activated dopamine system modulates peripheral immune cells, resulting in the expression of anti-inflammatory and neuroprotective cytokines in mice with ischemic stroke.

## Introduction

Ischemic stroke leads to motor impairment, which is a hallmark of Parkinson’s disease (PD)-like pathology observed in humans and experimental mouse models [1]. The striatum consists of GABAergic interneurons that express tyrosine hydroxylase (TH) [2]. These interneurons receive synaptic input from dopaminergic neurons located in the substantia nigra (SN) [3]. Borlongan et al. were the first to suggest that dopaminergic degeneration is relevant to stroke-induced changes in motor behavior [4]. This finding is further supported by additional studies demonstrating that stroke-induced striatal infarcts lead to the loss of TH+ fibers in the striatum, resulting in the death of TH+ dopaminergic neurons in the SN, a remote area connected to the primary ischemic lesion [5–8]. The degeneration of the striatonigral pathway, which disrupts the dopamine system, may contribute to PD-like pathology in stroke patients.

The importance of endogenous striatal dopamine transmission has been proven in PD patients with motor behavioral deficits [9, 10]. The supply of dopamine to injured striatal lesions is crucial for recovering motor function in patients with PD [11, 12]. In the clinic, the administration of levodopa (L-DOPA, l-3,4-dihydroxyphenylalanine), the precursor of dopamine, is used as the gold standard therapy for patients with PD [13–18]. Similar to those in PD patients, targeting striatal lesions displaying degeneration of the striatonigral pathway has also been considered in stroke patients. For example, emerging clinical studies have shown the effect of L-DOPA treatment on behavioral recovery in patients with ischemic stroke [13, 17, 19]. Long-term therapy with L-DOPA was beneficial for improving motor behavior in patients with chronic stroke [13]. This evidence suggests that targeting the dopamine system could be a potential therapy for stroke. However, it is unclear which mechanism is involved in the stroke-induced disruption of the dopamine system and how L-DOPA regulates striatonigral degeneration to improve motor recovery in ischemic stroke patients.

The accumulation of immune cells, such as microglia/macrophages and lymphocytes, orchestrates excessive immune and inflammatory responses in the ischemic brain by releasing inflammatory factors. Previous studies have demonstrated the role of L-DOPA as an immune modulator in PD [20, 21]. These studies suggest that the beneficial effect of L-DOPA on ischemic stroke may occur through the modulation of the immune response. Among various immune components, IL-13 is a well-known anti-inflammatory cytokine released by immune cells. Studies have highlighted the neuroprotective effects of IL-13 in ischemic stroke. Peripheral administration of IL-13, for example, reduced stroke-induced brain injury and improved sensory and motor functions in mice [22]. Another study also demonstrated that IL-13 improved long-term recovery in mice after ischemic stroke [23]. The beneficial effect of IL-13 has been implicated in modulating microglia/macrophages toward anti-inflammatory responses [23–25].

Previous studies have shown that dopamine can induce or inhibit IL-13, depending on the disease and the specific types of cells it affects. One study demonstrated that dopamine promotes the expression of IL-13 in cultured CD4 T cells, which is associated with allergic lung inflammation through the differentiation of T helper 2 cells [26]. In contrast, dopamine-reduced IL-13 expression in innate lymphoid cells (ILCs) plays a role in suppressing ILC2-driven allergic airway inflammation [27]. However, it is not clear how dopaminergic signaling regulates IL-13 in the context of ischemic stroke.

Based on the broad implications of L-DOPA in ischemic stroke and the immune response, we hypothesized that L-DOPA exerts motor recovery and neuroprotective effects on striatonigral degeneration after a stroke by modulating endogenous immune cells. To test this hypothesis, we investigated dopaminergic neuronal degeneration in the striatonigral pathway and motor behavior in a mouse model of stroke induced by transient middle cerebral artery occlusion (tMCAO). We further investigated the effect of L-DOPA on IL-13 and immune responses, providing detailed insight into the neuroprotective mechanism of L-DOPA in ischemic stroke.

## Results

### Stroke induces striatonigral degeneration

**Fig. 1:**
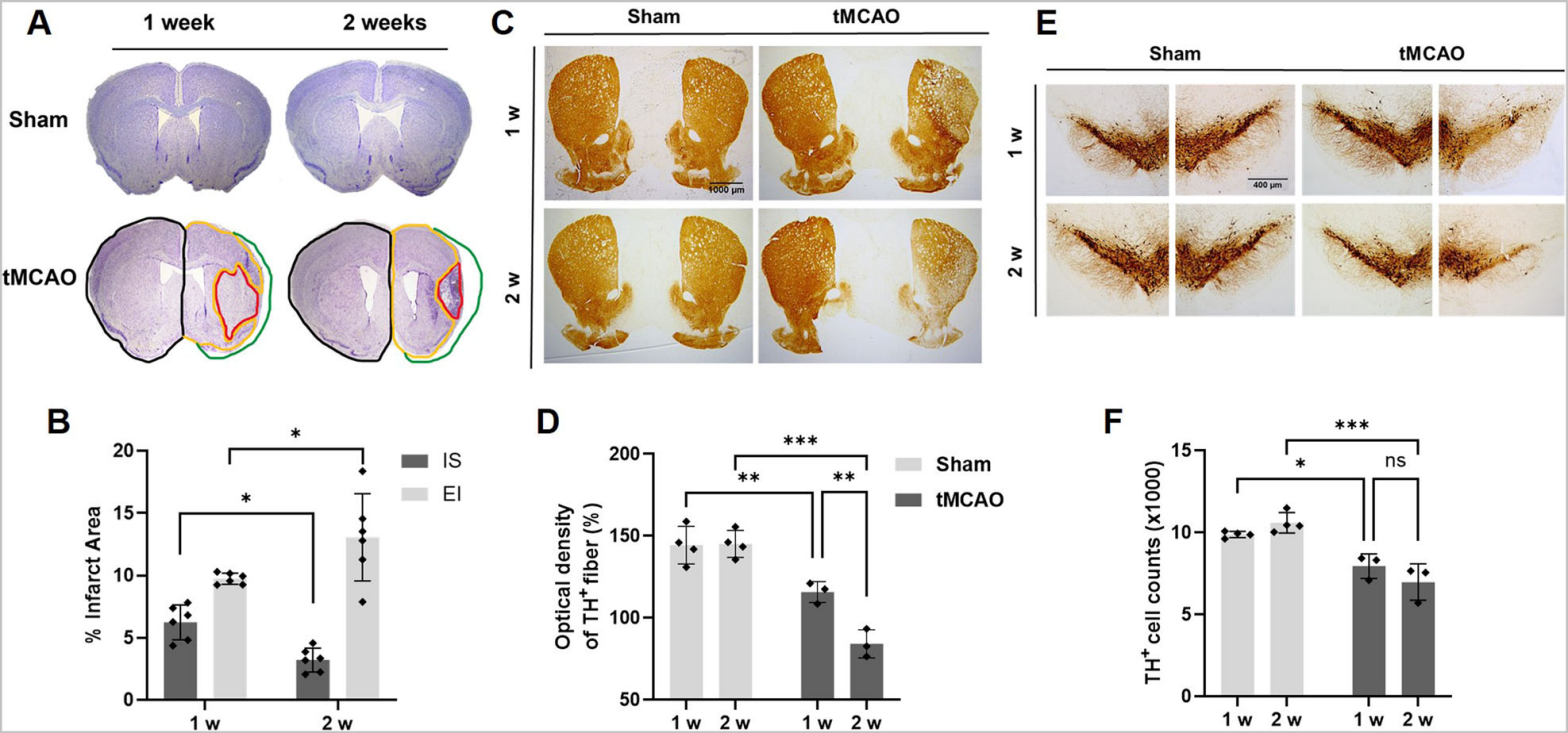
tMCAO induces degeneration of nigrostriatal dopamine neurons in vivo. **A** Representative cresyl violet-stained sections of sham and tMCAO mouse brains at 1 and 2 weeks after tMCAO. **B**. The graph shows the estimated infarct area (EI). The EI was calculated by subtracting the total ipsilateral volume from the total contralateral volume (black line) (namely, differences between the hemispheres, green). NI (noninjured tissue, yellow); IS (ischemic scar tissue, red); T (total ipsilateral tissue = NI + IS); Numbers of tMCAO mice at 1 week (*n* = 6) and 2 weeks (*n* = 6). **C**–**F** Photomicrographs of TH+ fibers in the striatum (STR) and TH+ cells in the substantia nigra (SN) at 1 week and 2 weeks after tMCAO are shown. The scale bars are 1000 μm (STR) and 400 μm (SN). The optical density of the TH+ fibers in the STR is presented. The number of TH+ cells in the SN is displayed. *p < 0.05, **p < 0.01, and ***p < 0.001 indicate a significant difference compared to the sham group. The mean ± SD is calculated by six pictures of each animal obtained from the sham (*n* = 4) and tMCAO (*n* = 3) groups. The difference in the *p*-value between the two groups was evaluated using the Student’s unpaired *t*-test. The staining intensity or the number of TH+ cells was quantified on seven slides per mouse. C contralateral, I ipsilateral.

We investigated whether stroke induces striatonigral degeneration in mice. Cresyl violet-stained brain sections showed extensive infarction in the mouse brain at 1 and 2 weeks (w) following tMCAO (Fig. 1A). In the subacute ischemic brain after a stroke, the affected area undergoes atrophy, leading to a decrease in brain volume. In this study, noninjured tissue (NI), ischemic scar tissue (IS), remaining total ipsilateral tissue (T = NI + IS), and resorbed tissue (estimated infarct: EI) were analyzed using a previously reported method [28]. Atrophy of the injured area was more significant at 2 w after the tMCAO, which is consistent with the previous reports (Fig. 1B) [29]. To investigate whether stroke induces striatonigral degeneration, we measured the presence of TH-positive (+) fibers and neuronal cells in the striatum and substantia nigra (SN) of the mouse brain following tMCAO. We observed a significant decrease in the optical density of TH+ fibers in the striatum at 1 w and 2 w post-tMCAO (Fig. 1C, D). Notably, the reduction in the TH+ fiber density was more pronounced at 2 w than at 1 w post-tMCAO. We also investigated whether tMCAO has a retrograde effect on TH+ neurons in the SN. Surprisingly, our results revealed a significant decrease in the number of TH+ neurons in the SN at 1 w and 2 w post-tMCAO (Fig. 1E, F). These findings demonstrate that ischemia in the forebrain striatum leads to the death of TH+ fibers in that area, which is primarily affected by ischemia. Additionally, it also leads to TH+ neuronal death in the SN.

**Fig. 2:**
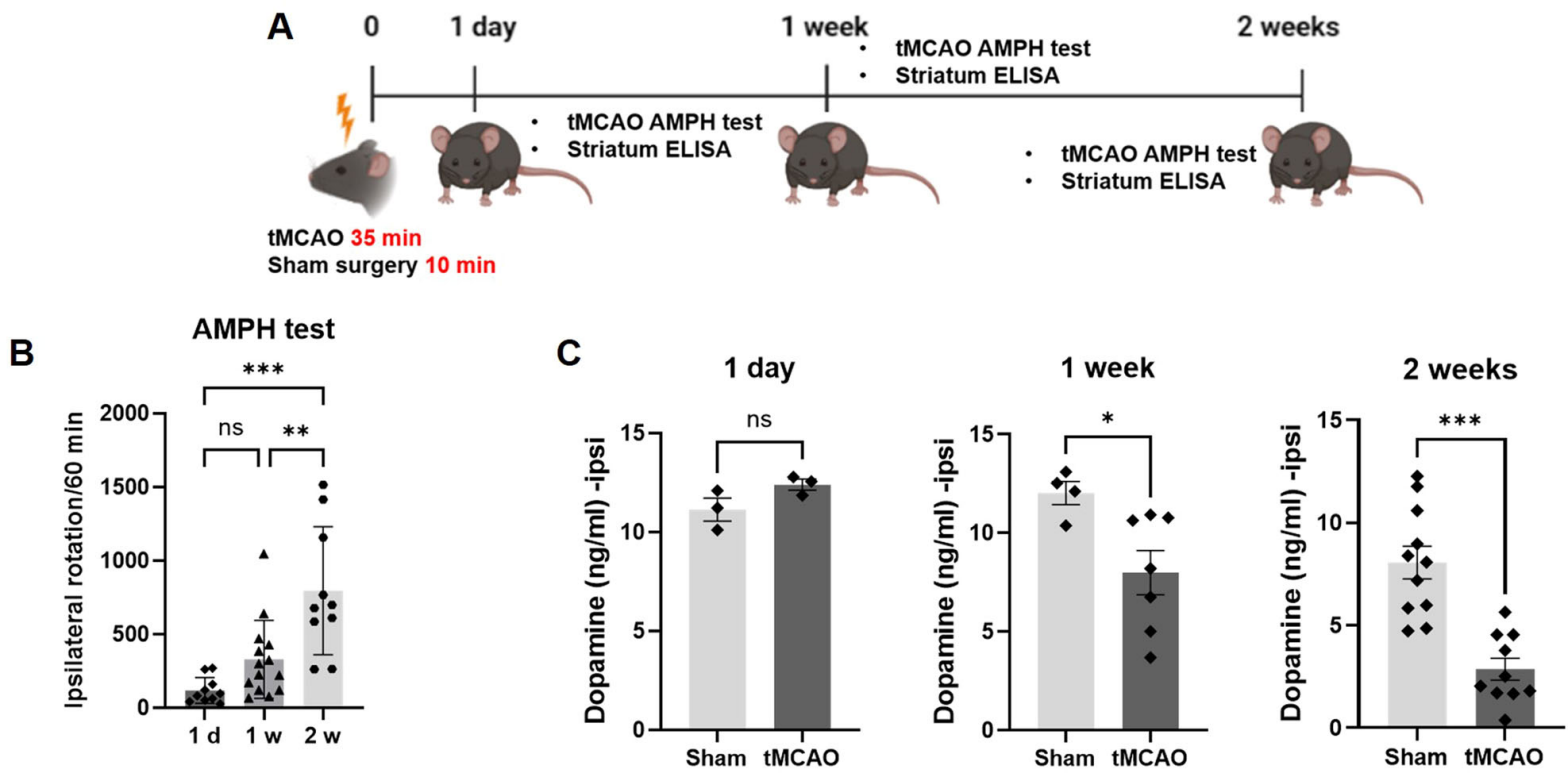
The amphetamine-induced rotation test and dopamine levels in the striatum. **A** Scheme illustrating the rotational testing in the striatum following amphetamine administration at 1 day, 1 week, and 2 weeks after tMCAO. **B** The results of the amphetamine-induced rotation test were recorded for 1 day (*n* = 10), 1 week (*n* = 14), and 2 weeks (*n* = 10). **C** The dopamine levels of the ipsilateral striatum were measured using ELISA. *p < 0.05, **p < 0.01, and ***p < 0.001 indicate statistically significant differences compared to the sham group. Mean ± SD: Sham (1 d, *n* = 5; 1w, *n* = 4; 2w, *n* = 11); tMCAO (1 d, *n* = 3; 1w, *n* = 7; 2w, *n* = 13). The statistical *p*-value between the two groups was evaluated using the Student’s unpaired *t*-test.

The striatum is the brain area that regulates and coordinates movement. To investigate whether stroke-induced striatonigral degeneration is associated with motor behavior, we conducted an amphetamine-induced rotation test (Fig. 2A). This test effectively determines the impairment in striatal lesions by showing imbalances in response to dopaminergic stimulation between the contralateral and ipsilateral hemispheres [30]. After tMCAO, we observed vigorous ipsilateral rotation at 1 w, which was not significantly different from that at 1 d after stroke (Fig. 2B). At 2 w post-tMCAO, the ipsilateral rotations of the mice were significantly greater than those at 1 d and 1 w post-tMCAO (Fig. 2B). These data indicate that there were more significant differences in response to dopaminergic stimulation between the contralateral and ipsilateral hemispheres at 2 w post-tMCAO. This finding also suggests a gradual increase in striatonigral degeneration on the ipsilateral side following stroke. Furthermore, we determined the loss of dopaminergic terminals by measuring dopamine levels on the ipsilateral side. There was a significant reduction in dopamine levels on the ipsilateral side at 1 w and 2 w post-tMCAO, negatively correlated with the rotation rate (Fig. 2C).

### L-DOPA treatment attenuates striatal neuron degeneration

**Fig. 3:**
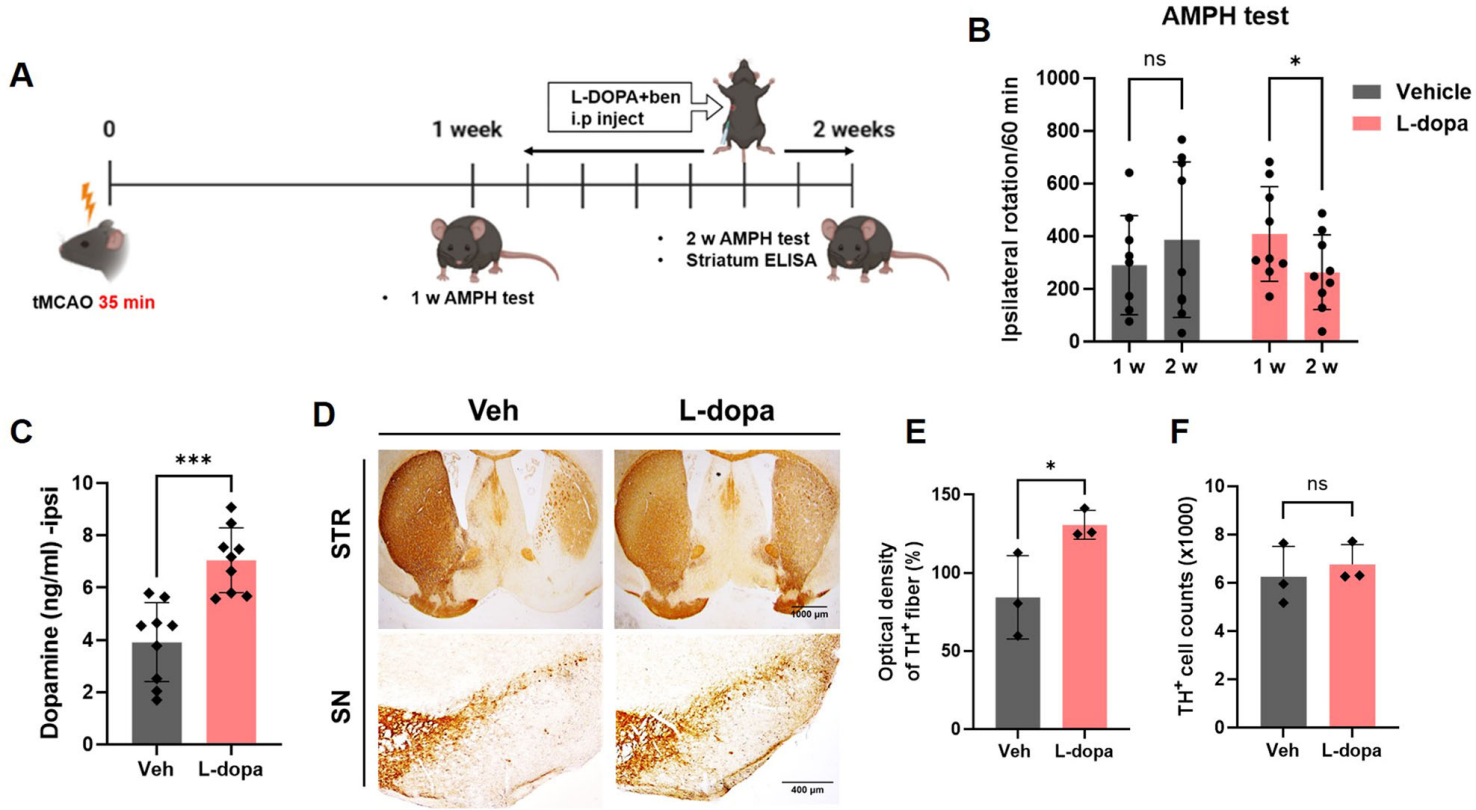
Effect of L-DOPA on tMCAO. **A** Scheme illustrating the administration of L-DOPA/benserazide and the amphetamine-induced rotation test. **B** Results of the amphetamine-induced rotation test at 1 and 2 weeks after treatment with L-DOPA/benserazide in tMCAO mice (*n* = 9 per group). **C** Measurement of dopamine levels in the ipsilateral striatum tissues by ELISA (*n* = 9–10 per group). **D** Photomicrographs of TH+ cells in the SN and TH+ fibers in the STR are shown for the vehicle and L-DOPA/benserazide groups 2 weeks after tMCAO. **E** The optical density of TH+ fibers in the STR is presented. **F** The number of TH+ cells in the SN is displayed. Each bar represents the mean ± SD. *p < 0.05 and ***p < 0.001 indicate statistically significant differences compared to the sham group. The statistical significance between the two groups was evaluated using the Student’s unpaired *t*-test.

We next tested whether L-DOPA treatment could improve stroke-induced motor deficits. Mice were treated with either vehicle or 20 mg/kg of L-DOPA by intraperitoneal injection based on previous studies [31–33]. Mice that exhibited ipsilateral rotation at 1 w post-tMCAO were randomly selected to receive either a vehicle or L-DOPA for 1 week (Fig. 3A). Remarkably, mice treated with L-DOPA exhibited reduced rotational behavior at 2 w compared to 1 w post-tMCAO (Fig. 3B). L-DOPA is a precursor to dopamine. We found that one week of L-DOPA treatment significantly increased dopamine levels in the ipsilateral brain (Fig. 3C). Consistent with the improved rotational behavior and increased dopamine levels, the optical density of TH+ fibers in the striatum significantly increased with L-DOPA compared to the vehicle treatment (Fig. 3D, E). However, the number of TH+ neurons in ipsilateral SN was not affected by L-DOPA treatment (Fig. 3D, F). These results suggest that L-DOPA treatment improves motor function by reducing the degeneration of striatal neurons, but it does not protect against the death of TH+ dopaminergic neurons in the SN.

### IL-13 is involved in L-DOPA-induced behavioral improvement

**Fig. 4:**
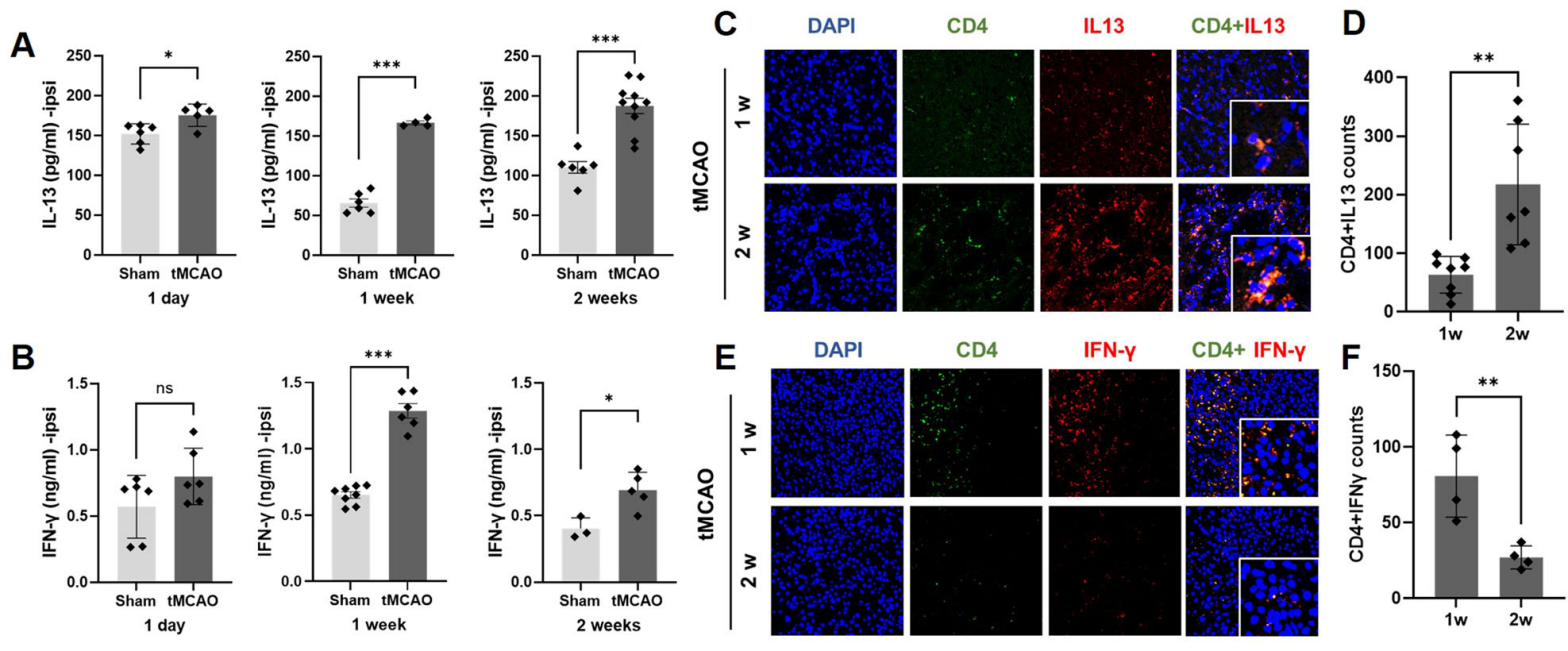
Expression of cytokines in the brain tissues after tMCAO. **A**, **B**. IL-13 and IFN-γ levels were measured in the ipsilateral striatum tissues by ELISA. IL-13 (Sham: 1 d, *n* = 6; 1w, *n* = 6; 2w, *n* = 6; tMCAO: 1 d, *n* = 5; 1w, *n* = 4; 2w, *n* = 10), IFN-γ (Sham: 1 d, *n* = 6; 1w, *n* = 8; 2w, *n* = 3; tMCAO: 1 d, *n* = 6; 1w, *n* = 6; 2w, *n* = 5). **C**, **D** Slides from frozen tissues were stained with anti-CD4 (green) and anti-IL-13 (red) antibodies. Fluorescence images of IL-13 in CD4 T cells were merged (yellow). IL-13-producing cells were counted in CD4 T cells in the striatum. Mean ± SD: IL-13 (tMCAO: 1w, *n* = 8; 2w, *n* = 7). **E**, **F** Slides from paraffin tissues were stained with anti-CD4 (green) and anti-IFN-γ (red) antibodies. Fluorescence images of IFN-γ in CD4 T cells were merged (yellow). IFN-γ-producing cells were counted in CD4 T cells in the striatum. Mean ± SD: IFN-γ (tMCAO: 1w, *n* = 4; 2w, *n* = 4). *p < 0.05, **p < 0.01, and ***p < 0.001 indicate a significant difference compared to the sham group. The statistical *p*-value between the two groups was evaluated using the Student’s unpaired *t*-test.

**Fig. 5:**
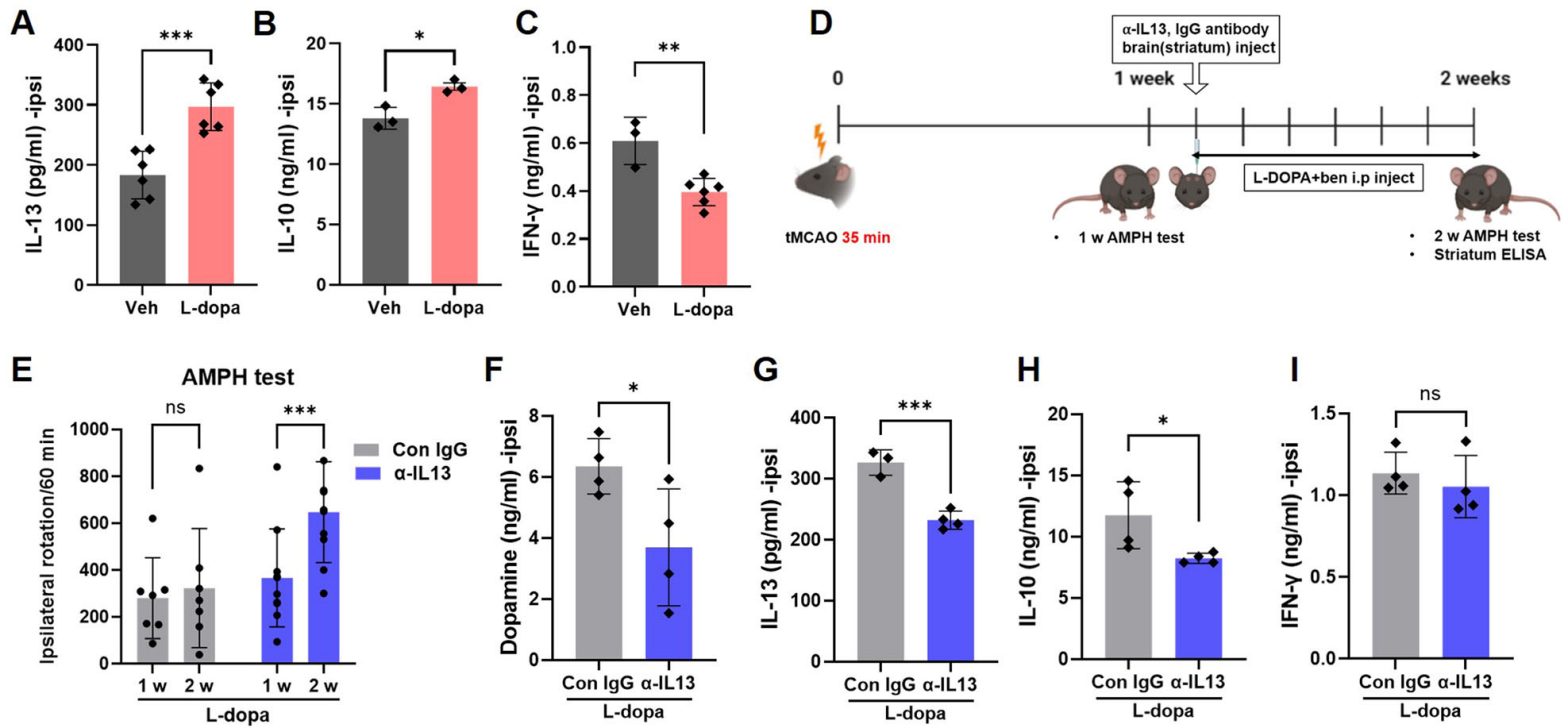
Effect of anti-IL-13 antibody on tMCAO. **A**–**C** Measurement of IL-13, IL-10, and IFN-γ levels in the ipsilateral striatum tissues of stroke mice treated with L-DOPA/benserazide was performed using ELISA (*n* = 3–6 per group). Each bar represents the mean ± SD. *p < 0.05, **p < 0.01, and ***p < 0.001 indicate a statistically significant difference compared to the vehicle control group. The statistical significance between the two groups was evaluated using the Student’s unpaired *t*-test. **D** Scheme of anti-IL-13 Ab and L-DOPA/benserazide injection and amphetamine-induced rotation. **E** The amphetamine-induced rotation test was conducted at 1 and 2 weeks after treatment with L-DOPA/benserazide and anti-IL-13 neutralizing Ab (1 μg/μL) in tMCAO (*n* = 7–10 per group). **F** Dopamine levels in ipsilateral brain tissues were measured using ELISA (*n* = 4 per group). **G**–**I** Levels of IL-13, IL-10, and IFN-γ in the ipsilateral striatum tissues were measured using ELISA (*n* = 4 per group). *p < 0.05, **p < 0.01, and ***p < 0.001 indicate a significant difference compared to the control IgG group. The result was expressed as the mean ± SD, and the statistical *p*-value between the two groups was evaluated using the Student’s unpaired *t*-test.

To investigate the potential involvement of IL-13 in the beneficial effects of L-DOPA in ischemic stroke, we assessed its expression in ischemic brain tissue. Using the same samples, we measured IFN-γ, an inflammatory cytokine regulated by L-DOPA in the ischemic brain [34]. Stroke significantly increased the levels of IL-13 and IFN-γ in the ischemic brain at 1 w and 2 w post-tMCAO (Fig. 4A, B). Our immunostaining data showed that IL-13 and IFN-γ were predominantly expressed in CD4 + T cells in the ischemic brain (Fig. 4C–F), whereas IL-4, IL-6, and IL-10 did not colocalize with CD4 + T cells (Supplementary Fig. 1). A few IL-13- or IFN-γ-expressing CD4 + T cells were observed in the sham control brain tissue (Supplementary Fig. 2). Interestingly, the level of IL-13 was more significant at 2 w post-tMCAO than at 1 w post-tMCAO, whereas the level of IFN-γ was decreased (Fig. 4C–F). These data suggest that CD4 + T cells are the primary source of IL-13 and IFN-γ in ischemic stroke at specific time points. In addition, L-DOPA further increased IL-13 and IL-10 levels (Fig. 5A, B) but significantly reduced IFN-γ levels in the ischemic brain (Fig. 5C). To investigate the role of IL-13 in the motor function improvement observed with L-DOPA treatment, we administered an anti-IL-13 antibody directly into the striatum one week after tMCAO, followed by L-DOPA treatment (Fig. 5D). Compare with mice treated with control IgG, mice treated with the anti-IL-13 Ab showed enhanced amphetamine-induced rotational behavior (Fig. 5E). Furthermore, anti-IL-13 Ab treatment reduced dopamine levels, as well as IL-13 and IL-10 levels, while IFN-γ levels remained unchanged in the ischemic brain (Fig. 5F–I). These data suggest that IL-13 is a critical regulator of L-DOPA-induced behavioral improvement.

### Induction of microglial phagocytosis by dopamine-induced IL-13

**Fig. 6:**
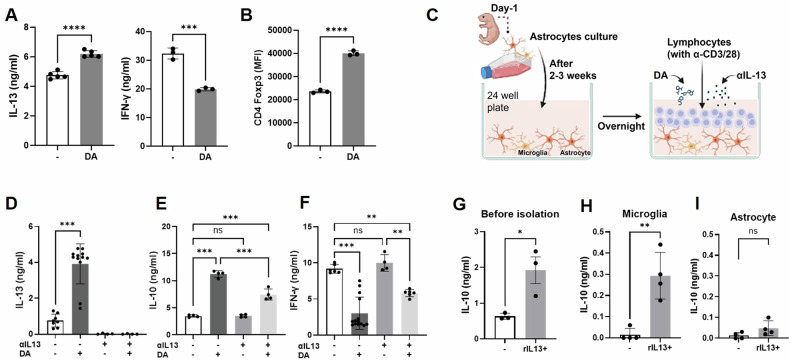
Effects of dopamine on IL-13 levels in vitro. **A**, **B**. CD4 T cells were stimulated with anti-CD3 and anti-CD28 antibodies (1 μg/mL) in the presence of 5 μg/mL of dopamine. After 48 h, IL-13 and IFN-γ levels in the culture media were measured using ELISA (*n* = 3 per group), and the level of Foxp3 expression was measured by flow cytometry. MFI: mean fluorescence intensity. **C** Scheme of a lymphocyte coculture system with astrocytes. **D**–**F** Lymphocytes were cocultured with astrocytes (including microglia) and stimulated with anti-CD3 and anti-CD28 antibodies (1 μg/mL) in the presence of dopamine and an anti-IL-13 neutralizing antibody (0.5 μg/mL). After 48 h, levels of IL-13, IL-10, and IFN-γ were measured using ELISA (*n* = 4–12 per group). **G**–**I**. The astrocyte culture contained ca. 20~30% of CD11b+ microglia. Therefore, microglia and astrocytes were purified with over 94% purity using MACS. The purified cell populations were then stimulated with recombinant IL-13 cytokines (20 ng/mL). After 24 h, IL-10 expression was measured in the culture media using ELISA (*n* = 3–4 per group). *p < 0.05, **p < 0.01, ***p < 0.001, and ****p < 0.0001 indicate a significant difference compared to the control group. The result was expressed as the mean ± SD, and the statistical *p*-value between the two groups was evaluated using the Student’s unpaired *t*-test. The comparison of multiple groups was evaluated using a one-way ANOVA with Tukey’s multiple comparison test. These experiments have been repeated at least two times.

We observed high expression of IL-13 and IFN-γ in CD4 + T cells in the ischemic brain and confirmed that L-DOPA treatment further regulated their expression (Figs. 4, 5). It has been reported that activated regulatory T cells (Tregs) secrete IL-13, which induces IL-10 production in macrophages [35]. To investigate the direct effects of dopamine on activated T cells, isolated CD4 T cells were stimulated with anti-CD3 and anti-CD28 antibodies in the presence of dopamine. Dopamine increased IL-13 but decreased IFN-γ expression in CD4 + T cells (Fig. 6A). At the same time, we observed increased expression of Foxp3, a marker of Tregs, in CD4 + T cells treated with dopamine (Fig. 6B). In addition, CD4 + CD25+ naïve Treg cells isolated from the spleen and lymph nodes were stimulated with anti-CD3 and anti-CD28 antibodies in the presence of dopamine. Interestingly, dopamine increased IL-13 production in CD4 + CD25+ cells (Supplementary Fig. 3). To further investigate whether dopamine regulates the expression of IL-13 and IFN-γ in the presence of astrocytes, we conducted a coculture experiment of activated lymphocytes with astrocytes (Fig. 6C). Primary astrocytes were cultured for 2–3 weeks and then seeded in a 24-well plate at a density of 2 × 10^5^ cells one day before being cocultured with lymphocytes. The next day, 1 × 10^6^ lymphocytes were added to the cultured astrocytes and activated with anti-CD3 and anti-CD28 Abs in the presence of dopamine. We observed increased IL-13 and IL-10 expression, while the level of IFN-γ was decreased by dopamine (Fig. 6D–F). Under anti-IL-13 Ab treatment, the dopamine-induced increase in the expression of IL-10 decreased, while the dopamine-induced decrease in the expression of IFN-γ was partially reversed (Fig. 6D–F). This finding suggested that the expression of IL-10 is partially dependent on IL-13. In our primary astrocyte culture, we observed approximately 20–30% microglial contamination (Supplementary Fig. 4). Therefore, we next determined which cells in the cultures responded to IL-13. Over 94% of the purified microglia and astrocytes were isolated separately and exposed to recombinant IL-13 protein (Supplementary Fig. 4, Fig. 6G–I). We observed a significant release of IL-10 in the culture media of microglia exposed to rIL-13, suggesting that microglia may respond primarily to IL-13 (Fig. 6H). These results suggest that dopamine induces IL-13 secretion in T cells, which in turn triggers the release of IL-10 in microglia. At the same time, dopamine reduces the expression of IFN-γ in T cells.

**Fig. 7:**
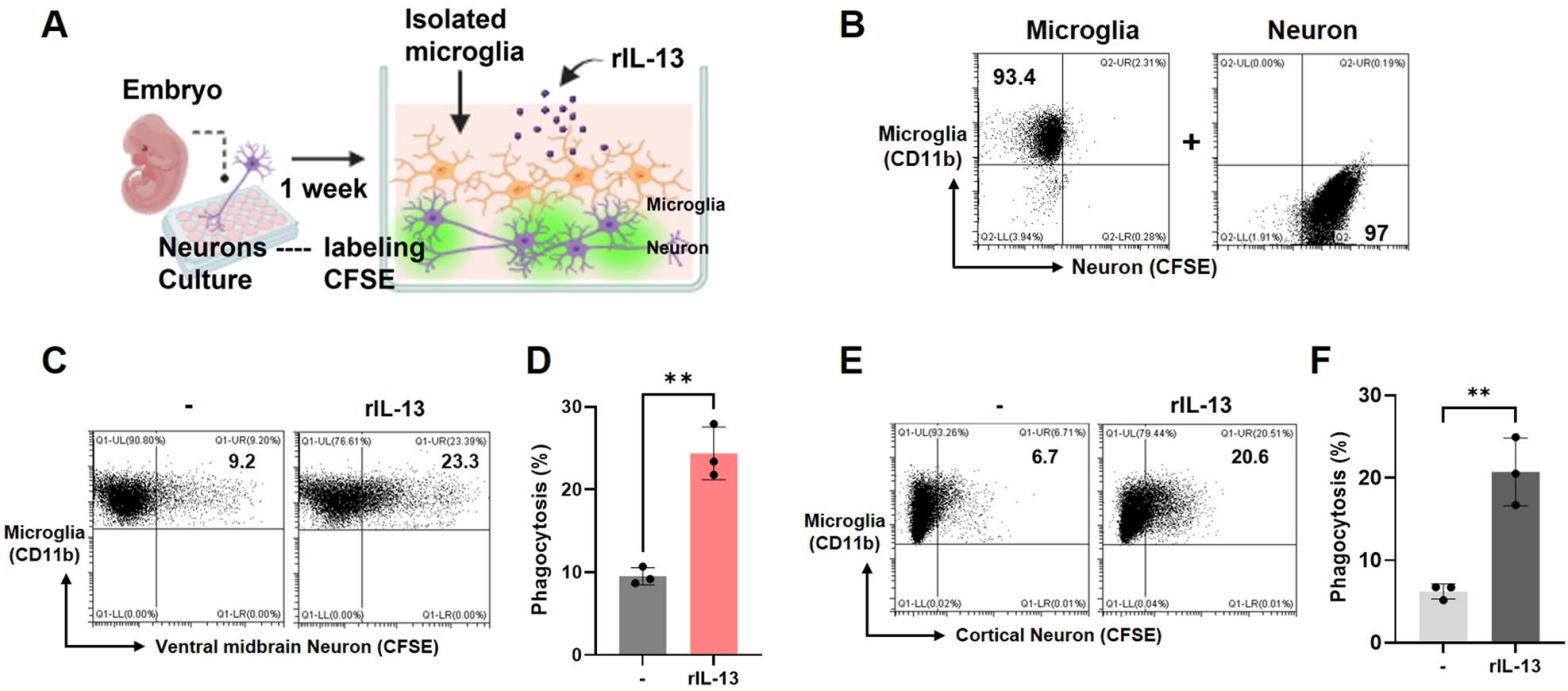
IL-13-induced phagocytic activity of microglia. **A** Schematic diagram illustrating the testing of microglia’s efficacy in phagocytosing apoptotic neurons. **B**–**F** Purified microglia were cocultured with CFSE-stained neurons. Primary dopaminergic (**C**, **D**) and cortical (**E**, **F**) neurons were stained with CFSE. Purified microglia were cocultured with neurons in the presence of rIL-13 (20 ng/mL). After 15 min (dopaminergic neurons) and 30 min (cortical neurons), microglia were stained with anti-CD11b antibodies, and the CFSE-positive cells indicating phagocytosis were analyzed using flow cytometry. (*n* = 3 per group). *p < 0.01 indicates a significant difference compared to the control group. The result was expressed as the mean ± SD, and the statistical *p*-value between the two groups was evaluated using the Student’s unpaired *t*-test. These experiments have been repeated at least two times. All schematic graphics were created using a subscription to BioRender.com.

It has been reported that IL-13 promotes macrophage efferocytosis during the resolution of inflammation [35]. To investigate the effect of T-cell-derived IL-13 on microglia, we hypothesized that IL-13 may stimulate microglial phagocytosis. We cultured primary mesencephalic dopaminergic neurons isolated from the ventral midbrain and stained them with CFSE, a fluorescent dye. These neurons were subsequently co-cultured with purified CD11b+ microglia in the presence of recombinant IL-13 protein (Fig. 7A, B). CD11b+ microglia were gated, and CFSE+ cells were analyzed using flow cytometry (Supplementary Fig. 5). Intriguingly, the number of CFSE-positive microglia was significantly increased by IL-13 treatment, suggesting that IL-13 increases the microglial engulf of the CFSE-stained neurons (Fig. 7C, D). Similar results were obtained with cortical neurons (Fig. 7E, F). These findings indicate that IL-13 enhances the phagocytic activity of microglia.

## Discussion

The beneficial effect of L-DOPA treatment on stroke-related motor behavior has been demonstrated in clinical and experimental rodent models [13, 17, 32]. However, the underlying mechanism by which L-DOPA improves motor behavior remains unclear. In the present study, we investigated the mechanism underlying the neuroprotective effect of L-DOPA on the striatonigral pathway in a mouse model of ischemic stroke. We aimed to understand how L-DOPA regulates inflammatory cytokines and immune cell functions.

L-DOPA is the neurotransmitter dopamine precursor and can potentially activate postsynaptic neurons, thereby promoting motor recovery [36, 37]. The efficacy of L-DOPA in treating PD has been demonstrated by stimulating the remaining intact postsynaptic neurons. Similarly, L-DOPA could target stroke-damaged striatal nerves and increase residual postsynaptic activity [19]. However, the effect of L-DOPA on striatal recovery has not been studied in ischemic stroke patients. In this study, we investigated the treatment effect of L-DOPA in ischemic stroke by examining the following outcomes: (1) improvement in motor behavior, as evidenced by a reduction in amphetamine-induced rotational behavior, and (2) increase in the optical density of TH+ fibers in the striatum. These results demonstrate that L-DOPA promotes motor recovery in ischemic mice by restoring striatonigral activity. This further supports the therapeutic potential of L-DOPA in stroke patients.

Intensive neuroinflammation occurs in the ischemic brain [33, 34]. In this study, we focused on the role of IL-13, an anti-inflammatory cytokine, as a mechanism for the beneficial effect of L-DOPA in ischemic stroke. The role of IL-13 appears to be diverse and controversial in several contexts. For example, IL-13 was detrimental to neuronal survival by enhancing neuroinflammation [38–42]. In PD, IL-13 activates the IL-13 receptor alpha 1 (IL-13Rα1) on dopaminergic neurons, increasing the susceptibility of the cells to damage caused by reactive oxygen species (ROS). IL-13Rα1 deficiency partially protected against inflammatory damage in PD mouse models [39, 43]. In contrast, some studies have highlighted the neuroprotective effects of IL-13. This is achieved by modulating the response of microglia/macrophages or inducing apoptosis in inflammatory microglia/macrophages [23–25]. Miao et al. showed that IL-13 ameliorates neuroinflammation and promotes functional recovery in traumatic brain injury (TBI) [44]. Another study demonstrated that IL-13 protects neurons from glutamate-driven excitotoxic cell death, suggesting a direct neuroprotective role in TBI [45]. However, these studies did not consider the effect of IL-13 in the inflammatory environment, such as the scenario involving immune cell infiltration. In the present study, we observed a significant increase in IL-13 expression in the subacute ischemic mouse brain after stroke (1 w and 2 w post-tMCAO) (Fig. 4A, C, D). Furthermore, this increase was further enhanced by L-DOPA treatment (Fig. 5A). By neutralizing IL-13, we confirmed that IL-13 mediated the improvement in behavioral function in ischemic mice treated with L-DOPA (Fig. 5E). Therefore, our results support the beneficial role of IL-13 in ischemic stroke.

The expression of IL-13 in the striatum has not yet been investigated in stroke. We observed extensive colocalization of IL-13 in CD4 + T cells in the striatum (Fig. 4C). Interestingly, we also found that IL-10, another anti-inflammatory cytokine, was regulated by IL-13 (Fig. 5H). Further in vitro coculture studies revealed that dopamine increased IL-13 expression in CD4 + T cells and induced Treg cells (Fig. 6A, B). In addition, we found that microglia exposed to IL-13 exhibited enhanced phagocytosis of CFSE-induced apoptotic neurons (Fig. 7 and Supplementary Fig. 5). These data suggest that L-DOPA-induced IL-13 produced by CD4+ Treg cells (and Supplementary Fig. 3) promotes the ability of microglia to resolve inflammation and facilitate wound healing. Similar results have been described in the presence of IL-13-producing Treg cells in atherosclerosis. Transfer of IL-13-producing Treg cells into mice with atherosclerosis improved lesional efferocytosis by promoting macrophage efferocytosis during the resolution of inflammation [35]. Treg cells play a crucial role in the resolution phase of inflammatory ischemic brain injury [46]. The polarization of IL-10-producing M2 macrophages or microglia is critical for clearing neuronal debris and minimizing brain damage after stroke [47–49]. Our previous study also demonstrated a correlation between the gradual recovery of the NSS in mice and the presence of Treg and IL-10-producing cells in the ischemic brain [29].

In contrast to IL-13 and IL-10, L-DOPA decreased the levels of IFN-γ, a proinflammatory cytokine, in the ischemic brain (Fig. 5C). The effects of dopamine on IFN-γ expression are still controversial. Several studies have shown that activation of the dopamine receptor D3 (D3R) facilitates the production of IFN-γ in CD4 + T cells [50, 51]. On the other hand, other studies have shown that dopamine inhibits already activated effector T cells, including the inhibition of IFN-γ secretion [52]. The effects of dopamine on T cells vary depending on the status of the T-cell type and the concentration of dopamine in vitro. This variation can be attributed to the differential expression of dopaminergic receptors on T cells [53, 54]. The effects of dopamine on T cells have been thoroughly reviewed in several articles [53, 55, 56].

Consistent with the in vivo results, our in vitro studies showed that L-DOPA dose-dependently decreased IFN-γ and IL-17A levels (Fig. 6F, Supplementary Fig. 6). The expression of IL-2 was dose-dependently increased by L-DOPA, whereas the levels of IL-4 and IL-6 were not affected (Supplementary Fig. 6). We observed a significant decrease in IFN-γ and IL-17A in cells treated with 5 and 10 μg/mL L-DOPA (25 and 50 μM), respectively (Supplementary Fig. 6). Dopamine at concentrations ranging from ~1 nM to 10 μM (10^−9^ to 10^−5 ^M) typically suppresses T cells that have already been activated by common T-cell activating molecules such as antigens, mitogens, and anti-CD3 and anti-CD28 antibodies. Josefsson et al. reported that dopamine, at high concentrations of 100 and 500 μM, inhibited mitogen (Con-A)-induced proliferation and cytokine production in mouse T cells by inducing apoptosis [52]. In our case, we observed cytotoxicity at a concentration of 20 μg/mL, which is approximately 100 μM. Therefore, we used a dose of dopamine that did not cause cytotoxicity in T cells in this study.

Our study observed that one week of treatment with L-DOPA significantly improved stroke behavior during the subacute phase (2 weeks after MCAO). While the beneficial effects of L-DOPA on stroke outcomes are consistent with previous clinical studies, our preclinical findings must be cautiously translated into clinical settings. In humans, recovery following a stroke typically occurs during the chronic phase, often necessitating prolonged L-DOPA treatment [13, 17, 19, 57–59]. However, L-DOPA treatment during the subacute phase—specifically, 5 to 10 days after a stroke—has not demonstrated beneficial effects [60]. This suggests that the critical timing for L-DOPA treatment does not align between human patients and our mouse models. The discrepancy may be partially attributed to (i) fundamental differences between humans and mice (e.g., age, metabolic rate, comorbidities) or (ii) the effectiveness of L-DOPA on motor recovery, which may depend on the stage of the disease. Therefore, we should carefully interpret our data to elucidate the relationship between chronic striatonigral degeneration and L-DOPA treatment in a clinical context. Further long-term animal studies utilizing various doses of L-DOPA should also be conducted.

In addition, we referenced studies on stroke by Ruscher’s group to determine the appropriate dosage of L-DOPA [32, 33]. They administered various doses of levodopa (1, 5, and 20 mg/kg) to intraperitoneal tMCAO rats. They observed significant improvements in sensorimotor function recovery and regulation of T cell-mediated immune responses at the 20 mg/kg dosage. Additionally, we considered a study by Perez-Pardo et al. that involved a model of PD [31]. In this study, PD mice were orally administered three doses of levodopa (5, 10, or 20 mg/kg) along with a diet that had therapeutic effects on PD, followed by motor function and spatial recognition assessments. Post hoc analyses revealed that the PD mice receiving the highest dose of levodopa (20 mg/kg) exhibited significantly better functional behavior than those treated with the lowest dose (5 mg/kg) or saline. However, several studies indicate that dyskinesia is a considerable side effect of long-term and high doses of L-DOPA treatment in PD [15, 16, 61–63]. Our study did not observe dyskinesia-like behavior in the mice following L-DOPA treatment, probably due to short treatment periods of 7 days. Nevertheless, strategies that reduce the risk of developing such side effects should be considered, such as modulating the L-DOPA treatment dose and period. Therefore, additional studies using various doses and treatment times of L-DOPA in ischemic stroke are necessary.

In summary, this study focused on the role of IL-13 as an endogenous regulator in L-DOPA-induced improvement of functional motor recovery and prevention of striatonigral degeneration in mice after an ischemic stroke. We demonstrate that exogenous L-DOPA increases IL-13 and IL-10 expression in the striatum. By conducting an in vitro coculture study, we confirmed that IL-13 secreted by CD4 T cells induces IL-10 expression in microglia. This induction may stimulate microglia/macrophages for phagocytosis and clearance of dead neurons in the ischemic brain. We implicate the role of IL-13 in the beneficial effects of L-DOPA on the survival of TH+ neurons survival and motor recovery in ischemic mice. These findings explain how L-DOPA reduces striatal degeneration in the ischemic brain and provide a valuable basis to support that L-DOPA treatment may be a beneficial therapeutic approach for stroke patients.

## Materials and methods

### Antibodies and chemicals

The data are listed in supplementary information Tables 1 and 2.

### Surgical procedure used for middle cerebral artery occlusion

Ten to twelve-week-old male C57BL/6 mice (Orient, Seongnam, South Korea) were used in this study. The mice were housed in a specific pathogen-free facility at Inha University in Incheon, Republic of Korea. All mice were provided with unlimited access to water and food throughout the entire experimental period. The laboratory temperature was maintained at 25 ± 2 °C, and the humidity was maintained at 60 ± 5%.

All animal experiments were approved by the Institutional Animal Care and Use Committee (INHA170908-513-5; INHA180420-558-4; INHA200518-701-7; INHA230525-875-2; INHA240723-939). Stroke was induced in mice by middle cerebral artery occlusion (MCAO) following a previously published method [29]. MCAO was induced using 6-0 nylon monofilament sutures (Doccol Corporation, Sharon, USA). During MCA occlusion, the mice were placed on a ventilator and kept under respiratory anesthesia with isoflurane (Kyungbo Pharmaceutical, Asan, Korea) mixed with 30% oxygen and 70% nitrogen. The concentration of isoflurane was adjusted to 2%. After placing the anesthetized mouse on its ventral side, an incision was made on the right side of the neck to expose the common carotid artery (CCA), internal carotid artery (ICA), and external carotid artery (ECA). After creating one knot in the CCA and two knots in the ECA and cutting between the two knots in the ECA, a nylon monofilament (filament size 6–0, diameter 0.09–0.11 mm, length 20 mm, coated tip diameter 0.25 ± 0.02 mm, 5–6 mm long, cat # 602556PK10Re, Doccol Corporation, Sharon, USA) was inserted into the ECA and then carefully placed into the ICA to obstruct blood flow in the MCA. To prevent drying of the surgical site, the area was coated with DPBS, and the filament was removed after being occluded for 35 min. After tying a knot at the incised ECA site, the knot on the CCA was released to restore blood flow, and then the incision site was sutured. Animals in the sham group underwent an identical procedure without MCAO. In the animal experiments, blinding was not employed.

### L-DOPA and benserazide treatment

Mice that exhibited ipsilateral rotations one week after tMCAO were randomly selected based on the distribution of behavioral scores, which were comparable among the groups. Mice were administered either a vehicle or L-DOPA for one week. L-DOPA (Levodopa; #PHR1271, Sigma‒Aldrich, MA, USA) and benserazide (#B0477000, Sigma‒Aldrich, MA, USA) were dissolved in sterilized water at concentrations of 20 mg/kg and 15 mg/kg, respectively. The mice were intraperitoneally injected daily with a 1 cc insulin syringe (324903, BD, NJ, USA) for 7 days, starting one week after tMCAO. For the vehicle group, an equal volume of sterile water was injected.

### Stereotaxic surgery for anti-IL-13 treatment

Mice that exhibited ipsilateral rotations one week after tMCAO were randomly selected based on the distribution of behavioral scores, which were comparable among the groups. Mice were anesthetized with chloral hydrate (360 mg/kg, administered via intraperitoneal injection) and then received either an anti-mouse IL-13 neutralizing antibody (α-IL-13 Ab; 1 μg/μL; R&D Systems) or control IgG (goat IgG; 1 μg/μL; R&D Systems) directly into the right striatum. [anteroposterior (AP) + 0.7 mm, mediolateral (ML) −2.8 mm, dorsoventral (DV) −5.0 mm at bregma] (Paxinos, 1998, Rat Brain in Stereotaxic Coordinates, 6th edition). It was injected at a rate of 1 µL/min into a syringe pump (KD Scientific, MA, USA) equipped with a 30-gauge beveled needle. After the injection, the needle was left in place for 10 min. The needle was then slowly withdrawn. After receiving a final intraperitoneal injection of L-DOPA and benserazide (Sigma‒Aldrich, MA, USA), the mice were euthanized by administering an overdose of isoflurane (Kyungbo Pharmaceutical, Asan, Korea), and their brains were harvested.

### AMPH-induced rotation test

The AMPH rotational test was performed as previously described [30, 64] to evaluate the behavioral impairment caused by the loss of the striatonigral pathway in tMCAO mice. Rotation data were collected over 60 min, starting 5 min after the intraperitoneal injection of D-amphetamine (2.5 mg/kg). The data are reported as the average net full 360-degree rotation per minute (ipsilateral minus contralateral rotation). The sham control mice did not exhibit any rotation (data not shown). In the L-DOPA injection group, AMPH was injected at least 30 min after the administration of L-DOPA. When the AMPH test rotation numbers in one week were significantly higher or lower than the normal distribution, they were excluded from the experiment.

### Cresyl violet staining

Mice were perfused transcardially with saline, followed by fixation using 4% paraformaldehyde (PFA; Biosesang, Seongnam, South Korea) dissolved in 0.1 M phosphate buffer. The brain tissues were fixed overnight in buffered 4% PFA at 4 °C and then stored in a 30% sucrose solution for 24 to 48 h at 4 °C. Subsequently, they were sliced into 30 μm-thick coronal sections using a cryotome FSE (Thermo Fisher Scientific, MI, USA). Tissue sections were stained with 0.1% cresyl violet acetate (Sigma‒Aldrich, St. Louis, MO, USA) dissolved in distilled water. The infarct area was measured using ImageJ software (NIH, Bethesda, MD, USA).

### TH staining

As described [65, 66], the mice were transcardially perfused with saline and then fixed using 4% PFA dissolved in 0.1 M phosphate buffer. The brain tissues were fixed overnight in 4% PFA at 4 °C and then stored in a 30% sucrose solution for 24 to 48 h. The tissue was sliced into 30 μm-thick coronal sections using a cryostat. After being preserved in 4% PFA, the brain tissues were embedded in paraffin to obtain 10 μm-thick sections. For the striatum (STR) and substantia nigra (SN) sections of the collected brain tissue, four evenly spaced sections were selected from anterior to posterior regions in six separate series of striatal and substantia nigra regions. Each selected section was subjected to DAB (3,3’-diaminobenzidine tetrahydrochloride hydrate, Sigma‒Aldrich, St. Louis, MO, USA) staining for anti-TH (1:2000, Pel-freeze, RO, USA). After staining, the sections were photographed using an Olympus BX43 microscope (Tokyo, Japan). The cell bodies expressing TH in the SN were counted using Image Gauge software (Fujifilm, Tokyo, Japan). At the same time, the optical density of TH+ fibers in the STR was measured using ImageJ software (NIH, Bethesda, MD, USA).

### Immunofluorescence staining

The brains were postfixed with 4% PFA. For the striatum sections, a blocking solution (0.3% Triton X-100, 1% BSA, 0.05% Tween 20, and 0.05% sodium azide in PBS) was applied and incubated for 1 h at room temperature (RT). The samples were incubated with the following primary antibodies: anti-CD4 (1:100, Invitrogen, CA, USA), anti-IFN-γ (1:100, R&D Systems, Minneapolis, MN, USA), and anti-IL-13 (1:100, R&D Systems, Minneapolis, MN, USA) overnight at 4 °C. The tissues were incubated with Alexa Fluor 488-conjugated anti-rat IgG (1:1000, Invitrogen, CA, USA) and Alexa Fluor 594-conjugate donkey anti-goat IgG (1:1000, Invitrogen, CA, USA) for 1 h. After the nuclei were stained with DAPI (1:1000, Invitrogen, CA, USA) for 20 min, VECTASHIELD Mounting Medium (Vector Laboratories, CA, USA) was applied to the slides. A coverslip was then placed on top for mounting. The CD4 + T cells expressing IL-13 or IFN-γ were visualized using a confocal microscope (U-TBI90, Olympus, Tokyo, Japan). The entire STR area was photographed at 20× magnification, and the positively stained cells were then quantified by counting. Three slides per mouse were examined. Similar results were obtained in two independent experiments.

### Primary astrocyte cultures

Primary astrocytes were obtained from newborn C57BL/6 mice aged 1–3 days [67]. The cortex without meninges was dissected and treated with 0.25% trypsin at 37 °C for 30 min. The cells were resuspended in a DMEM/F12 medium (Gibco, Massachusetts, USA) supplemented with antibiotics and 10% heat-inactivated fetal bovine serum (FBS, Gibco, Massachusetts, USA). The resuspended single cells were placed in T175 flasks and incubated at 37 °C with 5% CO_2_. After 2–3 weeks, we analyzed microglia using CD11b antibodies (BD Bioscience, NJ, USA) and astrocytes using ACSA-1 antibodies (Miltenyi Biotec, Germany).

### Primary cortical and mesencephalic dopaminergic neuron cultures

For the culture of dopaminergic neurons, the ventral midbrain of fetal ICR mice at embryonic day 13.5 was collected [68, 69]. For the culture of primary cortical neurons, cortical brains were obtained from fetal C57BL/6 mice on gestational days 13 to 14 [70, 71]. The tissue was suspended and filtered through a 70 μm nylon mesh to obtain a cell suspension. Cells (2 × 10^5^ cells/mL) were seeded in the poly-L-ornithine and laminin-coated 24-well plates at a volume of 500 μL per well and supplemented with 2 mM L-glutamine, 2% B-27 supplement, and 1% antibiotic-antimycotic (Gibco, Australia; Thermo Fisher Scientific, Massachusetts, USA). After 7 days, the cells were used for in vitro coculture with microglia.

### Isolation of lymphocytes and enrichment of CD4 + CD25 + T cells

RPMI 1640 (HyClone, GE Healthcare Life Sciences, PA, USA) was used as the medium for lymphocyte culture. The medium contained 0.1% 2-mercaptoethanol, 1% antibiotic-antifungal agent, and 10% FBS. To obtain single-cell suspensions, spleens and lymph nodes were isolated from 6 to 8-week-old female C57BL6 mice and placed in a 40 μm strainer on top of a 50 mL Falcon tube. The organs were compressed with a 1-mL syringe until fibrous tissue remained. After centrifugation, the red blood cells (RBCs) were removed using RBC lysis buffer (Sigma‒Aldrich, St. Louis, MO, USA). To isolate CD4 + CD25 + T cells from lymphocytes, we used a mouse CD4 + CD25+ regulatory T-cell isolation kit (Miltenyi Biotec, Germany). Although the purity of CD4 + CD25 + T cells was not high, the mean fluorescence intensity (MFI) of CD25 was much more significant in CD4 + CD25 + T cells than in CD4 + CD25- T cells (Supplementary Fig. 3). For the activation of T cells, 1 × 10^6^ isolated CD4 + CD25 + T cells were stimulated in a 24-well plate using plate-bound anti-CD3 antibodies (1 μg/mL) and soluble anti-CD28 antibodies (1 μg/mL). DA (dopamine, Sigma‒Aldrich, St. Louis, MO, USA) was added at a concentration of 5 μg/mL.

### Coculture of lymphocytes with primary astrocytes

We established a coculture system of lymphocytes with astrocytes. Primary astrocytes were cultured for 2–3 weeks and then seeded in a 24-well plate at a density of 2 × 10^5^ cells one day before being cocultured with lymphocytes. The next day, 1 × 10^6^ lymphocytes from the spleen and lymph nodes were added to the cultured astrocytes and incubated for 24 and 48 h in the presence of anti-CD3 and anti-CD28 antibodies (1 μg/mL). RPMI 1640 (HyClone, GE Healthcare Life Sciences, PA, USA) was used as the culture medium. The medium contained 0.1% 2-mercaptoethanol, 1% antibiotic-antifungal agent, and 5% FBS.

### Separation of microglia and astrocytes

We obtained 25–30% microglia in the astrocyte culture system (Supplementary Fig. 4). To isolate microglia, staining was performed using a PE-conjugated mouse anti-rat CD11b antibody (BD Biosciences, NJ, USA) for 15 min at 4 °C in the dark. After attaching anti-PE microbeads (Miltenyi Biotec, Bergisch Gladbach, Germany), microglia were separated using an LS column (Miltenyi Biotec). APC-conjugated GLAST (ACSA-1) was used to isolate astrocytes (Miltenyi Biotec). The purity of the isolated microglia and astrocytes was confirmed using flow cytometry (Cyto FLEX Flow Cytometer; Beckman Coulter, CA, USA). We achieved greater than 90% purity in isolating microglia and astrocytes (Supplementary Fig. 4). Isolated astrocytes and microglia were treated with mouse recombinant IL-13 (eBioscience, Massachusetts, USA) at a concentration of 20 ng/mL.

### Microglial phagocytosis

Primary neurons (2 × 10^5^) isolated from mouse embryos were cultured for seven days. Afterward, the culture medium was removed, and the neurons were labeled by staining them with CFSE (carboxyfluorescein succinimidyl ester) prepared in DPBS at a concentration of 1 μM for 20 min. Isolated microglia (2 × 10^5^) were added to primary neurons stained with CFSE. After 30 min, the population of phagocytes, consisting of CD11b + CFSE+ cells, was analyzed using flow cytometry (Supplementary Fig. 5).

### Cytokine and dopamine concentration analysis using enzyme-linked immunosorbent assay (ELISA)

The brain striatum tissues were weighed, minced into small pieces, and homogenized using a homogenizer in PBS supplemented with protease inhibitors (Roche, IN, USA). The experiments were conducted on ice with 10 μL of PBS per 1 mg of tissue. The homogenate was centrifuged at 5000 × *g* for 10 min to collect the supernatant. IL-13, IL-10, and IFN-γ levels in brain lysate extracts and culture media were measured following the manufacturer’s protocol (BD Biosciences, NJ, USA). The dopamine (DA) concentration was measured using the dopamine ELISA kit protocol provided by BioVision (MA, USA). The absorbance was measured at 450 nm using an automated plate reader (BioTek, Winooski, USA).

### Statistical analysis

Statistical analysis was performed using GraphPad Prism 9.5.1 (GraphPad Software, San Diego, CA, USA). *P*-values were calculated using Student’s unpaired *t*-test for statistical comparisons between the two groups. For the comparison of multiple groups, *p* values were evaluated using either one-way ANOVA or two-way ANOVA with Tukey’s multiple comparison test. The results are presented as the mean ± standard deviation (SD), and a *p-*value of *p* < 0.05 was considered to indicate statistical significance (*p < 0.05, **p < 0.01, ***p < 0.001, ****p < 0.0001).

## Supplementary information

Supplementary information

## Data Availability

All study data are included in the articles and/or Supplementary Figures and Tables.
