# Supplementary material for: Neuroprotective effect of L-DOPA-induced interleukin-13 on striatonigral degeneration in cerebral ischemia
Source: Cell Death Dis. 2024 Nov 22;15(11):854. doi: 10.1038/s41419-024-07252-x (PMC11584695; doi:10.1038/s41419-024-07252-x)
Supplement: Supplementary file 1 — Supplementary information [file 41419_2024_7252_MOESM1_ESM.pdf]

Neuroprotective Effect of L-DOPA-induced Interleukin-13 on Striatonigral Degeneration in Cerebral Ischemia

Eunhae Jeon[1,3,7], Myeong-Seong Seo[1,3,7], Enkhmaa Lkhagva-Yondon[1,3], Yu-Ree Lim[1,2], Seung-Woo Kim[2], Yu Jeong Kang[4], Jun Seok Lee[4], Byoung Dae Lee[4], Rayul Wi[4], So-Yoon Won[5], Young Cheul Chung[5], Eun S. Park[6*], Eunhee Kim[6*], Byung Kwan Jin[5*], and Myung-Shin Jeon[1,2,3*]

[1]Translational Research Center, Inha University Hospital, Incheon, Republic of Korea

[2]Department of Biomedical Sciences, College of Medicine, Inha University, Incheon, Republic of Korea

[3]Program in Biomedical Science & Engineering, Inha University, Incheon, Republic of Korea

[4]Department of Physiology, School of Medicine, Kyung Hee University, Seoul, Republic of Korea

[5]Department of Biochemistry & Molecular Biology, School of Medicine, Kyung Hee University, Seoul, Republic of Korea

[6]Vivian L. Smith Department of Neurosurgery, McGovern Medical School, The University of Texas Health Science Center at Houston, Houston, TX, USA

[7] These authors contributed equally: Eunhae Jeon, Myeong-Seong Seo

* Corresponding authors:

Myung-Shin Jeon (msjeon@inha.ac.kr), Translational Research Center, Inha University School of Medicine, B/308 Jungseok Bldg., 366 Seohae-daero, Jung-gu, Incheon 22332, Republic of Korea; Tel: +(82) 32 890-3682, Fax: +(82) 32 890-2462

Byung Kwan Jin (bkjin@khu.ac.kr), Department of Biochemistry & Molecular Biology, School of Medicine, Kyung Hee University, Seoul 02447, Republic of Korea; Tel: +(82)-2-961-9288, Fax: +(82)-2-969-4570

Eunhee Kim (Eunhee.kim@uth.tmc.edu), Vivian L. Smith Department of Neurosurgery, McGovern Medical School, The University of Texas Health Science Center at Houston, 6431 Fannin St. Houston, TX, 77030, USA: Tel: +1-713-500-6837

Eun S. Park (Eunsu.park@uth.tmc.edu), Vivian L. Smith Department of Neurosurgery, McGovern Medical School, The University of Texas Health Science Center at Houston, 6431 Fannin St. Houston, TX, 77030, USA: Tel: +1-713-500-5534

Supplemental Figure 1

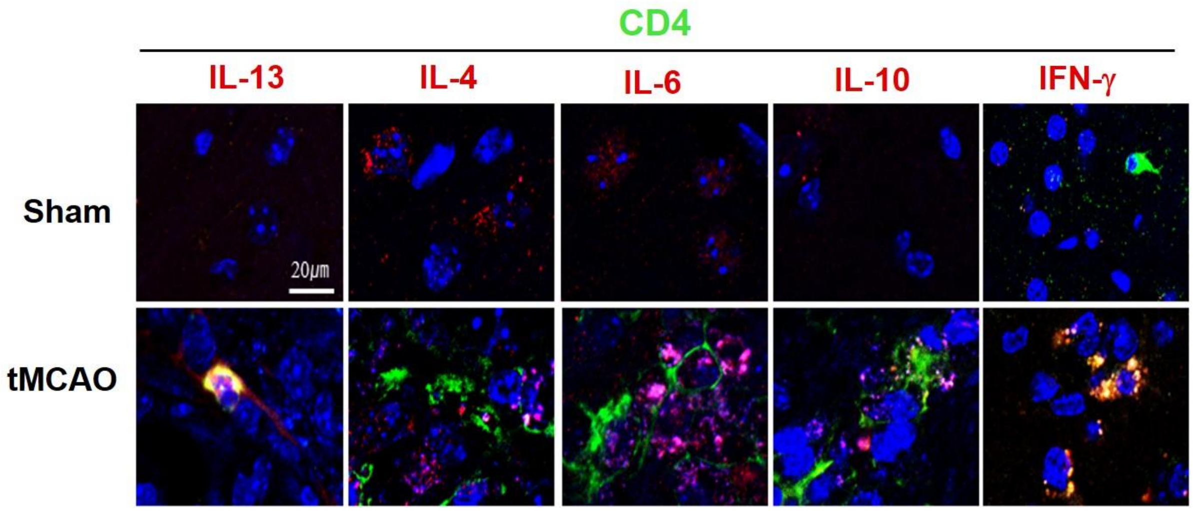

Supplementary Figure 1. Expression of cytokines in the brain tissues one week after tMCAO. One week after tMCAO, brain tissues were stained with antibodies for various cytokines (red) and CD4+ T cells (green). Fluorescence images of IL-13, IL-4, IL-6, IL-10, and IFN-γ in CD4+ T cells were merged (yellow).

Supplemental Figure 2

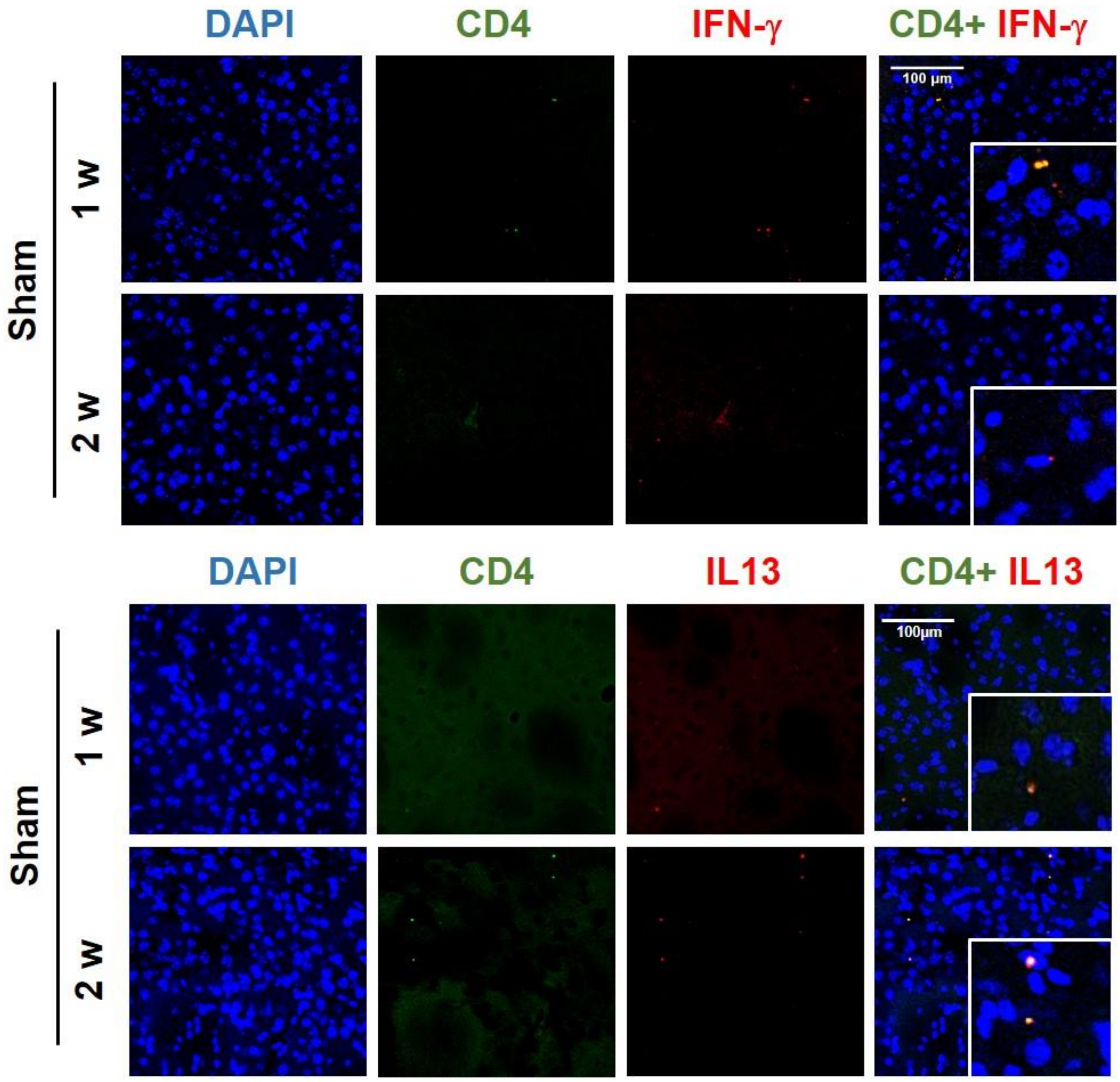

Supplementary Figure 2. Expression of IFN-γ and IL-13 in CD4 T cells in the striatum of Sham. Slides of frozen brain tissues from Sham mice were stained with anti-CD4 (green) and either IFN-γ or anti-IL-13 (red) antibodies. Fluorescence images of IL-13 or IFN-γ in CD4+ T cells were merged (yellow). (Sham 1w, n = 5; 2w, n = 6).

Supplemental Figure 3

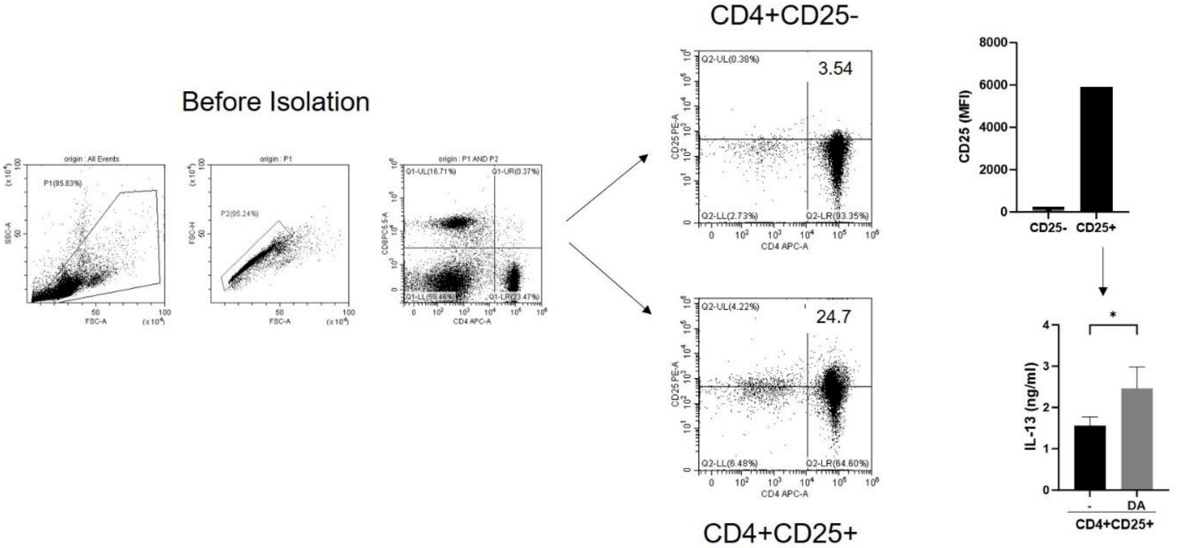

Supplementary Figure 3. IL-13 was increased in enriched CD4+CD25 T cells by dopamine. Lymphocytes were obtained from the spleen and lymph nodes of C57BL/6 mice. CD4+CD25+ T cells were purified using the MACS separation system and then stimulated with anti-CD3 and anti-CD28 antibodies (1 μg/mL) in the presence of dopamine (5 μg/mL). After 48 hours, the expression of IL-13 was measured in the culture media using ELISA. *: $p \leq 0.05$.

Supplemental Figure 4

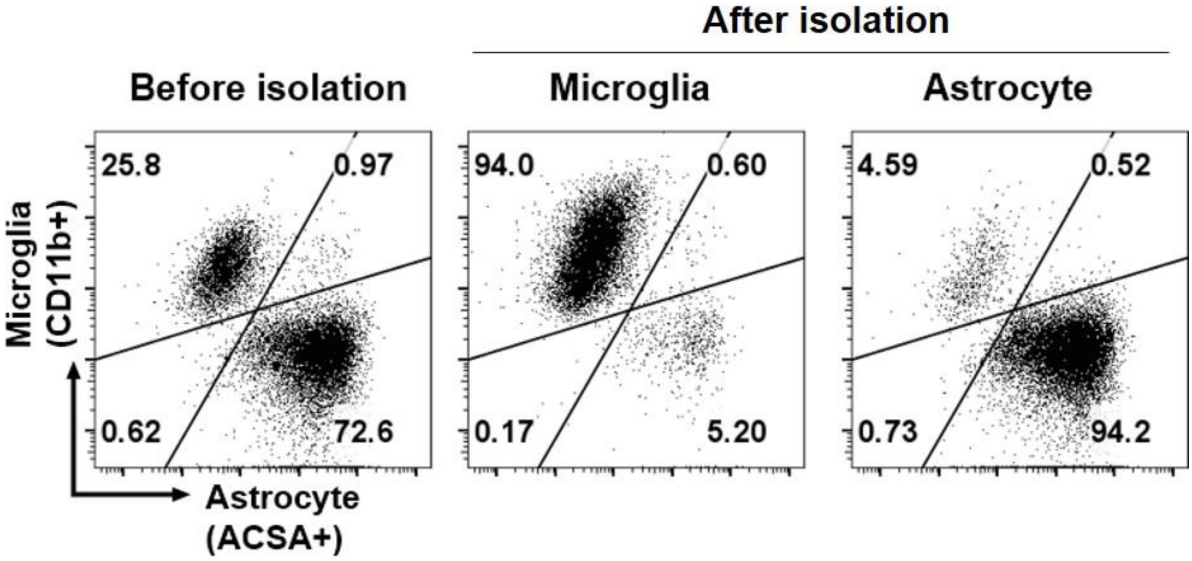

Supplementary Figure 4. Purification of microglia and astrocytes using the MACS system. Primary astrocytes were cultured for 2~3 weeks, the cells were stained with either CD11b-PE or ACSA-APC. Microglia or astrocytes were purified using anti-PE or anti-APC beads. The purity was over 90%.

Supplemental Figure 5

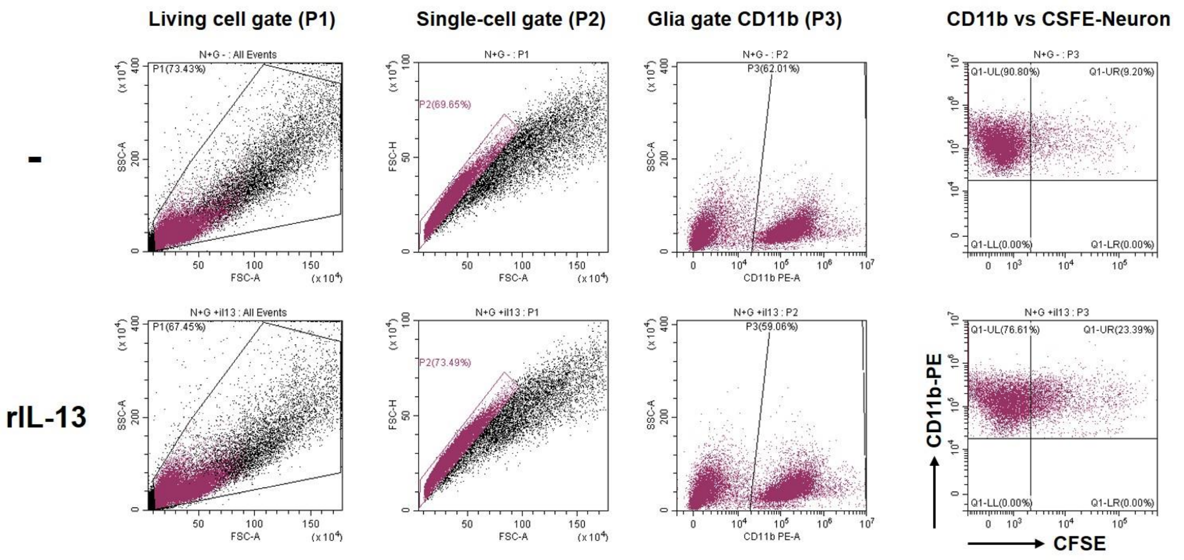

Supplementary Figure 5. Gate strategy for the analysis of phagocytic cells. Primary dopaminergic neurons from ICR mice were stained with CFSE fluorescence dye and co-cultured with purified microglia in the presence of rIL-13 (20 ng/mL). After 15 minutes, microglia were stained with anti-CD11b antibodies. Subsequently, the CFSE-positive cells, indicating phagocytosis, were analyzed using flow cytometry.

Supplemental Figure 6

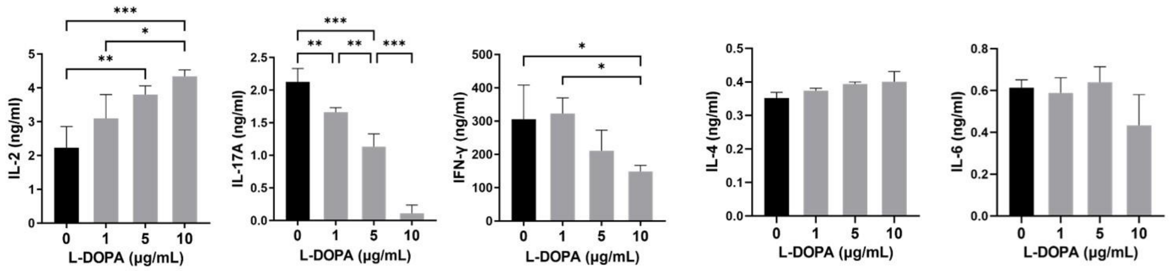

Supplementary Figure 6. Analysis of T-cell cytokines after L-DOPA treatment *in vitro*. Lymphocytes were isolated from lymph nodes and spleen and then stimulated with anti-CD3 and anti-CD28 antibodies (1 μg/mL). L-DOPA was treated at 1, 5, and 10 μg/mL concentrations. The levels of cytokines IL-2, IL-17A, IFN-γ, IL-4, and IL-6 were measured using ELISA after 48 hours of culture.; *: $p \le 0.05$; **: $p \le 0.01$; ***: $p \le 0.001$. The result was expressed as mean ± SD, and the statistical *p*-value between the two groups was evaluated using the Student's unpaired t-test. The comparison of multiple groups was evaluated using a one-way ANOVA with Tukey's multiple comparison test.

Supplementary Table 1. Antibodies

Antibodies & Kit	Source	Identifier	Application
Alexa Fluor 488 Rat IgG	Invitrogen (CA, USA)	A11006	IF (1/1000)
Alexa Fluor 594 Rabbit IgG	Invitrogen (CA, USA)	A11037	IF (1/1000)
Alexa Fluor 594 Goat IgG	Invitrogen (CA, USA)	A11058	IF (1/1000)
Alexa Fluor 594 mouse IgG	Invitrogen (CA, USA)	A-11005	IF (1/1000)
Annexin V Kit	BD Biosciences (NJ, USA)	556547	FACS (1/50)
Anti CD28	Biogems(CA, USA)	10312-25-1000	1 μg/mL
Anti CD3	Biogems(CA, USA)	05122-25-1000	1 μg/mL, (Coating (2 μg/mL)
Anti-human/mouse/Rat ACSA-1 APC	Miltenyi Biotec (Germany)	130-123-555	FACS (1/100)
Anti-tyrosine hydroxylase (TH)	Pel-freeze (RO, USA)	P40101-150	IHC (1/2000)
CD11b PE	BD Biosciences (NJ, USA)	553311	FACS (1/100)
CD25 APC	BD Biosciences (NJ, USA)	558643	FACS (1/200)
CD25 PE	BD Biosciences (NJ, USA)	553075	FACS (1/100)
CD4	Santa Cruz Biotechnology (TX, USA)	13573	IF (1/50)
CD4 APC	Biolegend (CA, USA)	100516	FACS (1/200)
Dopamine (DA) ELISA Kit	BioVision (MA, USA)	76212-074	ELISA
Goat anti-Rat IgG Biotinylated (H+L)	Vector Laboratory	BA-1000	IHC (1/400)
Goat IgG (Con-IgG)	R&D system (MN, USA)	AB-108-C	1μg/μL
IFN-γ Biotin	BD Biosciences (NJ, USA)	554410	ELISA
IFN-γ purified	BD Biosciences (NJ, USA)	551216	ELISA
IL-10 Biotin	BD Biosciences (NJ, USA)	554465	ELISA
IL-10 purified	BD Biosciences (NJ, USA)	551215	ELISA
IL-13 biotin	Invitrogen (CA, USA)	13-7135-85	ELISA
IL-13 purified	Invitrogen (CA, USA)	14-7133-85	ELISA
IL-17A Biotin	Biolegend (CA, USA)	507002	ELISA
IL-17A purified	Biolegend (CA, USA)	556902	ELISA
IL-2 Biotin	BD Biosciences (NJ, USA)	554426	ELISA
IL-2 Purified	BD Biosciences (NJ, USA)	554424	ELISA
IL-4	R&D system (MN, USA)	AF-504-NA	IF (1/400)
IL6	Santa Cruz Biotechnology (TX, USA)	SC-28343	IF (1/400)
IL-6 Biotin	BD Biosciences (NJ, USA)	554402	ELISA
IL-6 purified	BD Biosciences (NJ, USA)	554400	ELISA
IL-10	R&D system (MN, USA)	AF-217	IF (1/200)
Mouse IL-13 antibody	R&D system (MN, USA)	AF-413-NA	Surgery (1 μg/μL), IF (1/100), Culture (0.5 μg/mL)
Mouse/Rat IFN-γ antibody	R&D system (MN, USA)	AF-585-NA	IF (1/100)

Supplementary Table 2. Chemical lists

Name	Source	Identifier
Benserazide	Sigma (St Louis, MO, USA)	B0477000
CFSE (Carboxyfluorescein succinimidyl ester)	Invitrogen (CA, USA)	C34554
Cresyl violet	Sigma (St Louis, MO, USA)	C5042
DAB (3,3'-Diaminobenzidine tetrahydrochloride hydrate)	Sigma (St Louis, MO, USA)	D5637
DAPI	Invitrogen (CA, USA)	62248
Dopamine hydrochloride	Sigma (St Louis, MO, USA)	H8502
L-DOPA (Levodopa)	Sigma (St Louis, MO, USA)	PHR1271
Protease inhibitor cocktail	Roche (Indianapolis, IN, USA)	11697498001
Recombinant IL-13	eBioscience (MA, USA)	4-8131-62
